# eIF6 over-expression increases the motility and invasiveness of cancer cells by modulating the expression of a critical subset of membrane-bound proteins

**DOI:** 10.1186/s12885-015-1106-3

**Published:** 2015-03-15

**Authors:** Michela Pinzaglia, Claudia Montaldo, Dorina Polinari, Mattei Simone, Anna La Teana, Marco Tripodi, Carmine Mancone, Paola Londei, Dario Benelli

**Affiliations:** 1 Istituto Pasteur-Fondazione Cenci Bolognetti and Department of Cellular Biotechnologies and Haematology, Sapienza University of Rome, Via Regina Elena 324, 00161 Rome, Italy; 2 L. Spallanzani National Institute for Infectious Diseases, IRCCS, Via Portuense 292, 00149 Rome, Italy; 3 European Molecular Biology Laboratory, Meyerhofstrasse 1, Heidelberg, 69117 Germany; 4 Department of Life and Environmental Science, Polytechnic University of Marche, Via Brecce Bianche, 60131 Ancona, Italy

**Keywords:** Protein synthesis, Ribosome biogenesis, eIF6, cdc42, Cell migration

## Abstract

**Background:**

Eukaryotic Initiation factor 6 (eIF6) is a peculiar translation initiation factor that binds to the large 60S ribosomal subunits, controlling translation initiation and participating in ribosome biogenesis. In the past, knowledge about the mechanisms adopted by the cells for controlling protein synthesis by extracellular stimuli has focused on two translation initiation factors (eIF4E and eIF2), however, recent data suggest eIF6 as a newcomer in the control of downstream of signal transduction pathways. eIF6 is over-expressed in tumors and its decreased expression renders cells less prone to tumor growth. A previous work from our laboratory has disclosed that over-expression of eIF6 in transformed cell lines markedly increased cell migration and invasion.

**Methods:**

Here, we performed a quantitative proteomic analysis of membrane-associated proteins in A2780 ovarian cancer cells over-expressing eIF6. Differentially expressed proteins upon eIF6 overproduction were further investigated *in silico* by Ingenuity Pathway Analysis (IPA). RT-qPCR and Western blot were performed in order to validate the proteomic data. Furthermore, the effects of a potent and selective inhibitor ML-141 in A2780 cells were evaluated using transwell migration assay. Finally, we explored the effects of eIF6 over-expression on WM793 primary melanoma cell lines.

**Results:**

We demonstrated that: (i) the genes up-regulated upon eIF6 overproduction mapped to a functional network corresponding to cellular movements in a highly significant way; (ii) cdc42 plays a pivotal role as an effector of enhanced migratory phenotype induced upon eIF6 over-expression; (iii) the variations in abundance observed for cdc42 protein occur at a post-transcriptional level; (iv) the increased cell migration/invasion upon eIF6 over-expression was generalizable to other cell line models.

**Conclusions:**

Collectively, our data confirm and further extend the role of eIF6 in enhancing cell migration/invasion. We show that a number of membrane-associated proteins indeed vary in abundance upon eIF6 over-expression, and that the up-regulated proteins can be located within a functional network controlling cell motility and tumor metastasis. Full understanding of the role eIF6 plays in the metastatic process is important, also in view of the fact that this factor is a potentially druggable target to be exploited for new anti-cancer therapies.

**Electronic supplementary material:**

The online version of this article (doi:10.1186/s12885-015-1106-3) contains supplementary material, which is available to authorized users.

## Background

Protein synthesis and ribosome biogenesis are the most expensive processes for the cell in terms of energy and biosynthetic precursors. Cells are able to respond rapidly to the changes of the surrounding environment, modifying the expression profile of existing mRNAs and controlling the rate of ribosome biogenesis at any given time through multiple regulatory mechanisms.

Favorable stimuli (growth factors or nutrients) up-regulate ribosome, and consequently protein synthesis, to ensure enhanced growth and proliferation [1,2]. In contrast, stress circumstances down-regulate ribosome biogenesis reducing protein synthesis and cell proliferation [3]. Taken together, ribosome biogenesis and translational control are critical processes that are inextricably linked to cell growth and proliferation, permitting the cells to respond quickly to altered environmental conditions.

Increased cell proliferation, which is also a common characteristic of a perturbed cell cycle in cancerous cells, requires a general increase in protein synthesis that is, in many cases, sustained also by up-regulation of the ribosome biogenesis rate. Extensive studies focused on signal transduction pathways, such as PI3K-AKT-mTOR and RAS-MAPK, showed that their deregulation affects the function and expression of various components of the translational machinery, thus modifying the expression of specific mRNAs at the level of protein synthesis [4,5]. Hence, translation factors and ribosomal proteins impaired in their expression were recognized as a consequence of cancer progression and interpreted as a result of the higher biosynthetic demand of cycling cells [6]. However, during the last two decades, increasing data suggest an active role of ribosome biogenesis and translation factors in tumorigenesis. For example, the mere over-expression of the translation initiation factor eIF4E has been widely recognized to be sufficient to transform cells, regulating the preferential expression of specific proteins or the general translation rate [7,8]. Similarly, numerous genetic diseases harbouring mutations in distinct components involved in ribosome biogenesis, collectively referred as “ribosomopathies”, are prone to developing cancer [9]. In this perspective, the molecular mechanisms involved in protein synthesis represent a cause of cancer progression instead of a consequence.

One of the translation factors recently demonstrated to have a role in the control of protein synthesis and aberrantly expressed during cancer is the eukaryotic initiation factor 6 (eIF6) [10,11]. This is an essential protein that is expressed differently in various tissues and at different developmental stages. Although the mechanism whereby eIF6 acts in tumorigenesis is still not understood, it has been established to be rate-limiting for cell growth and transformation both in *in vitro* and *in vivo*. Indeed, eIF6 haploinsufficient mice are less susceptible to Myc and growth factor-induced tumors [12].

eIF6 is a conserved 25 kDa protein present in eukaryotes and archaea with a high grade of similarity [13]. It was initially identified as an anti-association factor in wheat germ [14] for its ability to bind the 60S ribosomal subunits and thus prevent their association with the 40S ribosomal subunits to form the 80S initiation complex. Differently, by the other translation initiation factors involved in the regulation of the first step of protein synthesis, eIF6 also exerts a role at the level of ribosome biogenesis. Indeed, genetic and biochemical experiments performed in yeast reclassified Tif6 (eIF6 homologue) as a ribosome biogenesis factor since it localizes in the nucleolus associated with pre-60S subunits and its loss produces a decrease of 60S particles [15].

A previous work from our laboratory [16] has disclosed that eIF6 transcription is under the control of the transmembrane receptor Notch-1, a protein involved in a wide variety of human neoplasms [17]. Inhibition of Notch-1 signaling in ovarian cancer cells by γ-secretase inhibitors slowed down cell-cycle progression and decreased the level of eIF6 protein. Remarkably, over-expression of eIF6, both in stably and transiently transfected cell lines, had little or no effect on cell proliferation but markedly increased cell migration and invasion, suggesting that eIF6 could be an important downstream effector whereby Notch-1 modulates cell motility in physiological or pathological conditions. Indeed, it has been known for some time that certain translational factors, notably eIF4E, are downstream targets of various signaling pathways that control cell migration, and its over-expression is causative of cancer progression [18].

The aim of the present study was to analyze the variations of protein abundance and composition caused by up-regulated eIF6 levels that could justify increased cell migration. By combining a stable-isotope labeling with amino acids in cell culture (SILAC), quantitative proteomic approach of cells over-expressing eIF6, computational analysis of proteomic data sets and molecular analysis we demonstrated that: (i) cells over-expressing eIF6 show a changed expression of a number of proteins; (ii) the proteins which appear to be up-regulated upon eIF6 overproduction mapped to a functional network corresponding to cellular movements in a highly significant way; (iii) cdc42, one of these proteins, plays a pivotal role as an effector of enhanced migratory phenotype induced upon eIF6 over-expression; (iv) the variations in abundance observed for cdc42 protein occur at a post-transcriptional level; (v) the increased cell migration/invasion upon eIF6 over-expression was generalizable to other cell line models.

## Methods

### Ethics statement

The use of the human derived cell cultures has been approved by the ethics committee of the Sapienza University of Rome, Italy, according to the ethical guidelines of the 1975 Declaration of Helsinki.

### Cell culture and treatments

The human ovarian cancer cells A2780 and human melanoma cell lines WM793 were cultured in RPMI 1640 medium (Gibco) supplemented with 10% FBS (Gibco), 1 mmol/L L-glutamine, 100 u/mL penicillin, and 100 ug/mL streptomycin in 5% CO_2_ incubator at 37°C. All cells were tested to ensure that there was no mycoplasma contamination. For the SILAC experiments, A2780 cells were cultured in “light” (^12^C_6_^14^ N_4_-arginine and ^12^C_6_-lysine, SILANTES) and “heavy” (^13^C_6_^15^ N_4_-arginine and ^13^C_6_-lysine, SILANTES) conditions for eleven passages before the next experiments. This period lasted about 4 weeks, where the SILAC “heavy” cells’ labeling was complete. SILAC labeling and proteomic analysis were performed twice.

For protein stability analysis, A2780 cells transfected with pcDNA3.1 and pcDNA3.1/eIF6 were treated 24 h after transfection with CHX (Sigma-Aldrich) at 40 μM for the indicated hours.

### Transfection assays

A2780 cells seeded in 60 mm or 100 mm dishes were transiently transfected at 80% confluence with 10 μg and 20 μg of the appropriate amount of plasmid, respectively. Lipofectamine 2000 reagent (Invitrogen) was employed according to the manufacturer’s instructions. Whenever required, ten times less of the pEGFP plasmid was used as reporter in order to detect the transfection efficiency. After 48 h of growth cells were lysed and subjected to the subsequent required analysis. The transfection of WM793 cell lines was performed in similar conditions.

For SILAC experiments, labeled A2780 cells were seeded in 100mm dishes and, once reached 80% confluence, the light labeled cells were transiently transfected with 10 μg/dish of human full-length eIF6 expression vector while the heavy labeled cells were transfected with the same amount of the control plasmid. pEGFP plasmid was also transfected at 1 μg/dish in both differentially labeled cell populations as control of transfection. Each transfection was performed in triplicate.

After 7 hours from transfection, cells were splitted and left to grow overnight in the respective light and high fresh medium. The next day GFP expression was analyzed by fluorescence microscopy and the transfections with efficiency higher than 60% were taken in account for next analysis.

### Membrane protein digestion, peptide purification and nanoLC analysis

For SILAC samples preparation, all cells were lysed and membrane proteins were isolated following the Membrane Protein Extraction Kit (M-PEK) protocol (CALBIOCHEM). Samples were analyzed by Bradford assay to determine the protein concentration. Equal amounts (200 μg) of membrane proteins from A2780/CTR and A2780/eIF6 cell lines were mixed and subsequently separated on 4 − 12% gradient gels (Invitrogen), stained by Simply Blue Safe Stain staining and visualized. Sixteen sections of the gel lane were cut. Protein-containing gel pieces were washed with 100 μL of 0.1 M ammonium bicarbonate (5 min at RT). Then, 100 μL of 100% acetonitrile (ACN) was added to each tube and incubated for 5 min at RT. The liquid was discarded, the washing step repeated once more, and the gel plugs were shrunk by adding ACN. The dried gel pieces were reconstituted with 100 μL of 10 mM DTT/0.1 M ammonium bicarbonate and incubated for 40 min at 56°C for cysteine reduction. The excess liquid was then discarded and cysteines were alkylated with 100 μL of 55 mM IAA/0.1 M ammonium bicarbonate (20 min at RT, in the dark). The liquid was discarded, the washing step was repeated once more, and the gel plugs were shrunk by adding ACN. The dried gel pieces were reconstituted with 12.5 ng/μL trypsin in 50 mM ammonium bicarbonate and digested overnight at 37°C. The supernatant from the digestion was saved in a fresh tube and 100 μL of 1% TFA/30% ACN were added on the gel pieces for an additional extraction of peptides. The extracted solution and digested mixture were then combined and vacuum centrifuged for organic component evaporation. Peptides were resuspended with 40 μL of 2.5% ACN/0.1% TFA, desalted and filtered through a C18 microcolumn ZipTip, and eluted from the C18 bed using 10 μL of 80% ACN/0.1% TFA. The organic component was once again removed by evaporation in a vacuum centrifuge and peptides were resuspended in a suitable nanoLC injection volume (typically 3–10 μL) of 2.5% ACN/0.1% TFA. An UltiMate 3000 nano-LC system (Dionex, Sunnyvale, CA) equipped with an integrated nanoflow manager and microvacuum degasser was used for peptide separation. The peptides were loaded onto a 75 μm I.D. NanoSeries C18 column (Dionex, P/N 160321) for multistep gradient elution (eluent A 0.05% TFA; eluent B 0.04% TFA in 80% ACN) from 5 to 20% eluent B within 10 min, from 20 to 50% eluent B within 45 min and for further 5 min from 50 to 90% eluent B with a constant flow of 0.3 μL/min. After 5 min, the eluted sample fractions were continuously diluted with 0.5 μL/min a-cyano-4-hydroxycinnamic acid (CHCA) and spotted onto a MALDI target using a Probot (LC-Packings/Dionex) with an interval of 20 s resulting in 144 fractions for each gel slice.

### Protein identification and quantification

MALDI-TOF-MS spectra were acquired using a 4800 Plus MALDI TOF/TOF Analyzer (AB Sciex, Foster City, CA). The spectra were acquired in the positive reflector mode by 20 subspectral accumulations (each consisting of 50 laser shots) in an 800 − 4000 mass range, focus mass 2100 Da, using a 355 nm Nb:YAG laser with a 20 kV acceleration voltage. Peak labeling was automatically done by 4000 Series Explorer software Version 3.0 (AB Sciex) without any kind of smoothing of peaks or baseline, considering only peaks that exceeded a signal-to noise ratio of 10 (local noise window 200 m/z) and a half maximal width of 2.9 bins. Calibration was performed using default calibration originated by five standard spots (ABI4700 Calibration Mixture). Only MS/MS spectra of preselected peaks (out of peak pairs with a mass difference of 6.02, 10.01, 12.04, 16.03, and 20.02 Da) were integrated over 1000 laser shots in the 1 kV positive ion mode with the metastable suppressor turned on. Air at the medium gas pressure setting (1.25 × 10 − 6 Torr) was used as the collision gas in the CID off mode. After smoothing and baseline subtractions, spectra were generated automatically by 4000 Series Explorer software. MS and MS/MS spectra were processed by ProteinPilot Software 2.0.1 (AB SCIEX) which acts as an interface between the Oracle database containing raw spectra and a local copy of the MASCOT search engine (Version 2.1, Matrix Science, Ltd.). The Paragon algorithm was used with SILAC (Lys + 6, Arg + 10) selected as the Sample Type, iodacetamide as cysteine alkylation, with the search option “biological modifications” checked, and trypsin as the selected enzyme. MS/MS protein identification was performed against the Swiss-Prot database (number of protein sequences: 254757; released on 20070123) without taxon restriction using a confidence threshold of 95% (Proteinpilot Unused score ≥1.31). The monoisotopic precursor ion tolerance was set to 0.12 Da and the MS/MS ion tolerance to 0.3 Da. The minimum required peptide length was set to 6 amino acids; two peptides were required for protein identification.

For quantitation, the Heavy/Light average ratio for a protein was calculated by ProteinPilot Software with automatic bias correction. Quantitation was based on a two-dimensional centroid of the isotope clusters within each SILAC pair. Ratios of the corresponding isotope forms in the SILAC pair were calculated, and lines fitting these intensity ratios gave the slope as the desired peptide ratio. To represent the ratio of a peptide being quantified several times, the median value was chosen. To minimize the effect of outliers, protein ratios were calculated as the median of all SILAC pair ratios that belonged to peptides contained in this protein. The percentage of quantitation variability was defined as the standard deviation of the natural logarithm of all ratios used for obtaining the protein ratio multiplied by a constant factor of 100. Only relative Heavy/Light (or Light/Heavy) ratios exceeding factor 1.5 were considered.

### Data analysis

Differentially expressed proteins were analyzed using Ingenuity Pathway Analysis (IPA, Ingenuity Systems; see www.ingenuity.com). The over-represented biological processes, molecular functions, and canonical pathways were generated based on information contained in the Ingenuity Pathways Knowledge Base. Right-tailed Fisher’s exact test was used to calculate a p-value determining the probability that each biological function and/or disease involved in that proteome profile alteration is due to chance alone.

### Western blot analysis

Total protein extract was obtained by lysing the cells with extraction buffer (20 mM Tris-HCl pH7.5, 150 mM NaCl, 1 mM EDTA pH 8.0, 1% Triton-X) and protease inhibitor cocktail (Roche). The protein concentration of A2780/eIF6 and control cell lysates was measured. Equivalent amounts of proteins from whole cell extracts or membranous fractions were denatured in a 5X sample loading buffer by heating at 95°C for 5 min and resolved by 15% SDS-PAGE. Proteins were electrotransferred to 0,45 μm nitrocellulose membrane (Amersham Biosciences) using a transfer apparatus according to the manufacturer’s protocols (Bio-Rad). After incubation with 5% nonfat milk in TBST (10 mM Tris, pH 8.0, 150 mM NaCl, 0,1% Tween 20) or with 3% BSA in TBST for 60 min, the membranes were washed once with TBST and incubated with antibodies against eIF6 (1:3000, BD Biosciences), cdc42 (1:1000, Cell Signaling), GAPDH (1:5000, Calbiochem Merck), Calnexin (1:200, Santa Cruz) or tubulin (1:20000, Sigma-Aldrich) at 4°C for 16 h. Membranes were washed once for 10 min and incubated with a 1:15000 dilution of horseradish peroxidase-conjugated anti-mouse or anti-rabbit antibodies for 1 h. Membranes were washed with TBST three times for 10 min each and developed with the ECL system (Amersham Biosciences) according to the manufacturer’s protocols. The intensity of the signals was quantified by densitometry analysis using ImageJ software.

### RNA extraction, reverse transcription and quantitative real-time PCR

Total RNA was extracted from ovarian or melanoma cancer cells using Trizol reagent (Invitrogen, Carlsbad, CA) following the manufacture’s protocol. cDNA was synthesized from 2 μg of total RNA using enhanced avian reverse transcriptase (Sigma-Aldrich). Quantitative real time PCR was performed with iCycler (Bio-Rad, Hercules, CA) on 2 μl of 1: 4 cDNA using 10 μl of SensiMix SYBR & Fluorescein Kit 2000 (Bioline). Cycling parameters were: 95°C for 10 min, followed by 40 cycles of 95°C for 15 s, 60°C for 1 min, 72°C for 10s. The relative amount of each mRNA was obtained by 2-ΔΔCt method and normalized to human housekeeping gene glyceraldehyde phosphate dehydrogenase (GAPDH) mRNA expression. The quantification of cdc42 mRNA in heavy fractions collected by sucrose gradients was performed by the coapplication-reverse transcription protocol adapted to that described elsewhere [19]. Specifically, cDNA was synthesized from 1 μg of total RNA using enhanced avian reverse transcriptase (Sigma-Aldrich) in presence of 0,8 μM oligo-(dT) primers and 2,5 μM of 18S-RNA-specific primer (5′-GAGCTGGAATTACCGCGGCT-3′). Quantitative real time PCR was performed with iCycler (Bio-Rad, Hercules, CA) on 1 μl of 1: 10 cDNA according to the above-described method.

Primer sequences used for cdc42 detection were as follows, sense: 5′-CCCGGTGGAGAAGCTGAG-3; and antisense: 5′-CGCCCACAACAACACACTTA-3′. For Hax1 detection, sense: 5′- GACCTCGGAGCCACAGAGAT-3′, and antisense: 5′-GGTGCTGAGGACTATGGAAC-3′. For HGF detection, sense: 5′- CAATAGCATGTCAAGTGGAG-3′; and antisense: 5′-CTGTGTTCGTGTGGTATCAT3′. For SDC1 detection, sense: 5′- AGGACGAAGGCAGCTACTCCT-3′, and antisense: 5′- TTTGGTGGGCTTCTGGTAGG-3′. For GAPDH detection, sense: 5′-AGCCACATCGCTGAGACA-3′, and antisense: 5′-GCCCAATACGACCAAATCC-3′. For rRNA detection, sense: 5′-TACCACATCCAAGGAAGGCAGCA-3′, and antisense: 5′- TGGAATTACCGCGGCTGCTGGCA-3′.

### Rac1/Cdc42 activity assays

Cdc42 activity was assessed using GST-tagged p21 binding domain of PAK1 (GST-PBD) according to the manufacturer’s instructions (Cell Signaling). Briefly, cells grown to ~70-80% confluence in regular growth medium following 24 h from transfection with pcDNA3.1 and pcDNA3.1/eIF6 constructs were collected in lysis buffer plus 1 mM PMSF. 500 μg of cleared extracts were incubated over-night at 4°C with glutathione beads coupled with GST-PBD to pull down GTP-bound cdc42. The amount of total and activated cdc42 was determined by Western blotting according to the above-described method.

### Migration assay

A2780 cells were pretreated in complete medium containing the molecular probe ML 141 for 24 h before plating (2.5 × 10^5^ per well) in the BD Falcon™ Cell Culture Inserts (BD Biosciences). Mock treatments were carried out pretreating the cells in the same medium with DMSO 0,1%. The chambers with the cells were placed on 24 well plates containing medium without serum plus the molecular probe at the same concentration of starting, or DMSO 0,1%. After 48 hours, cells migrated in the lower chamber were stained with crystal violet dye. In the lower chamber, medium supplemented with 10% FBS was used as chemoattractant and also in this chamber the molecular probe was added at the concentration used in the upper chamber. Experiments were carried out in triplicate and repeated three times. Membrane filters were imaged with ImageJ software.

For the experiments designed to evaluate the activity of ML 141 on eIF6-induced cell migration A2780 cells were transfected with the plasmid pcDNA3.1/eIF6 and the corresponding control according to that described above. After 24 hours pcDNA3.1/eIF6 and pcDNA3.1 A2780 cells were pretreated in complete medium containing the molecular probe ML 141 for 24 h before plating (2.5 × 10^5^ per well) in the BD Falcon™ Cell Culture Inserts (BD Biosciences) for the next 24 hours. Successively, the chambers with the cells were placed on 24 well plates containing medium without serum plus the molecular probe at the same concentration of starting. After 48 hours, cells migrated in the lower chamber were stained with crystal violet dye. In the lower chamber, medium supplemented with 10% FBS was used as chemoattractant and also in this chamber the molecular probe was added at the concentration used in the upper chamber. Experiments were carried out in triplicate and repeated three times. Membrane filters were imaged with ImageJ software.

To test the results of eIF6 over-expression on the migratory activity of the WM793 cells we adopted the same protocol described above in absence of ML 141 inhibitor.

### Invasion in matrigel-coated chambers

WM793 cells were transfected with the plasmid pcDNA3.1/eIF6 and the corresponding control according to as described above. After 24 hours, 2.5 × 10^5^ cells were seeded in the BD Matrigel invasion chambers (BD Biosciences). Cells were seeded in the upper chamber in medium without serum. After 24 hours, cells migrated in the lower chamber were stained with crystal violet dye. In the lower chamber, medium supplemented with 10% FBS was used as chemoattractant. Experiments were carried out in triplicate and repeated three times.

### Cell viability

A2780 cells were seeded into 35 mm plates at a density of 2 × 10^5^ per well and treated with the following: vehicle control (DMSO 0,1%), and 10 μM ML 141. The cells were treated for 24 h or 48 h. Cell viability was determined by trypan blue dye exclusion assay. Cells and growth medium were separately collected and Trypan Blue stained the dead cells in each fraction. The viable and unstained cells were counted. Triplicate wells of viable cells for each concentration were counted on a hemacytometer after trypsinization. Each well had three repeats of counting. The experiment was repeated three times.

### Immunoflurescence analysis

After 7 hours from transfections, cells in 60 mm or 100 mm dishes were spit and an adequate amount of resuspended cells were transferred in 35 mm dishes. The next day, when confluence was about 50%, cells in 35 mm dishes were washed 3 times with phosphate-buffered saline 1X (PBS) and fixed by adding 250 μL 4% paraformaldehyde (in PBS) for 15 min at RT. Then paraformaldehyde was removed, cells were washed 3 times with PBS and microscope slides were gently placed on cells for microscope examination. Transfection efficiency was calculated as the ratio of GFP-expressing cells over the total.

### Polysomal profiles

A2780 cells transfected with pcDNA3.1 and pcDNA3.1/eIF6 were treated 24 h after transfection with CHX (Sigma-Aldrich) to a final concentration of 100 μg/ml and then incubated at 37°C for 15 min. After washing the monolayer once with ice-cold PBS 1X + CHX (50 μg/ml), the cells were scraped in 500 μl of ice-cold lysis buffer (10 mM Tris-HCl pH 7.4, 10 mM KCl, 15 mM MgCl_2_, 1 mM DTT, 1% Triton-X 100, 1% deoxycholate, 0.5 units μl^-1^ rRNasin, 100 μg/ml CHX ) on ice. Cell debrises were removed by a 8 min centrifugation at 10,000 g at 4°C. 6 A_260_ units of supernatants were layered on top of a linear 15-50% (w/v) sucrose gradient containing 20 mM Tris-HCl pH 7.4, 5 mM MgCl_2_, 140 mM KCl, 0.5 mM DTT and 0.1 mg/ml CHX. The gradients were centrifuged at 4°C in a SW41 Beckman rotor for 3 h at 39,000 rpm and unloaded while monitoring absorbance at 254 nm with the EM-1 Econo UV absorbance instrument. Fractions (0.5 ml) were collected in 18 tubes and precipitated with an equal volume of isopropanol and 2 μl of GlycoBlue™ Coprecipitantat 15 mg/ml (Invitrogen) at -20°C over night. Successively, the samples were centrifuged at 13000 rpm for 30 min at 4°C. The resulting pellets were resuspended in 40 μl of DEPC-treated dH_2_O. The presence of the ribosomes in each fraction was checked analyzing 10 μl of each fraction onto 0,8% agarose gel. The fractions ribosome-free were pooled together and renamed “light fractions” whereas the fractions containing the ribosomes were pooled together and renamed “heavy fractions”. The total RNA of the last two fractions resulting from each cell sample was purified from the proteins with the Total RNA Purification Kit (Norgen Biotech Corp.) and quantified. The amount of cdc42 mRNA in each fraction was analyzed on equal amounts of RNA by qRT-PCR according to the above-described method.

## Results

### eIF6 over-expression perturbs the membrane proteome profiles of cultured ovarian cancer cells

**Figure 1:**
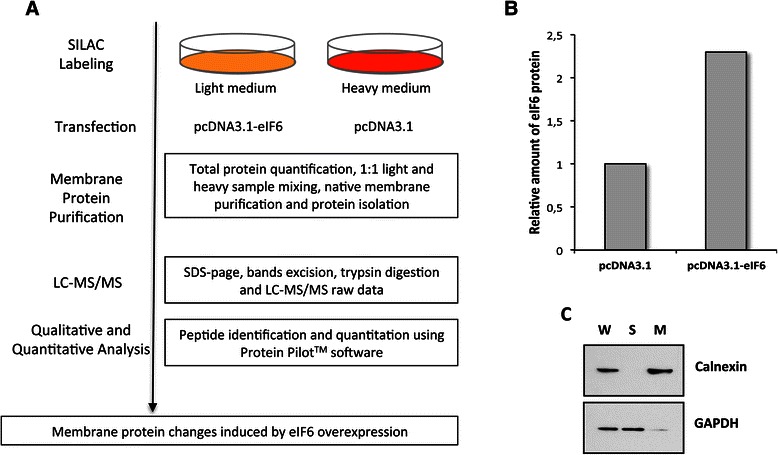
**SILAC-based proteomic analysis of membrane protein changes induced by eIF6 overexpression. A)** Schematic representation of SILAC-based proteomic workflow. **B)** 10 micrograms of protein whole cell extracts isolated from A2780 transfected either with pcDNA3.1 and pcDNA3.1-eIF6 were separated by SDS-PAGE and transferred to a PVDF membrane. Bands relative to eIF6 and tubulin (loading control) were detected with respective antibodies and analyzed by densitometry using Quality-One software (Bio-Rad laboratories, Richmond, CA). The X-axis shows the relative intensity of eIF6/tubulin; one representative experiment out of three is shown. **C)** Equal amounts of protein whole cell extracts isolated from control (pcDNA3.1) and eIF6-overexpressing (pcDNA3.1-eIF6) cells were mixed and subjected to native membrane purification. 10 micrograms of whole cell extract (WCE), soluble (S) and membrane (M) fractions were analyzed by western blotting. Antibodies against calnexin and GAPDH were used as markers of membrane and soluble fractions, respectively. One representative experiment out of three is shown.

As mentioned above, in a previous publication we observed that the principal effect of eIF6 over-expression in A2780 ovarian cell lines consisted in their increased motility/invasiveness. Independent of cell type and mode of migration, cell motility and invasiveness occur mainly through cytoskeletal remodeling and active participation of different protein complexes present on the cytoplasmic membrane at the front of the cells. Therefore, to identify the protein effectors of cell membranes through which eIF6 induces increased migration, we performed a membrane proteomic analysis of A2780 cells over-expressing eIF6 with respect to the control cells transfected with the empty vector. In particular, we applied the SILAC strategy that allows for quantitative comparisons among different samples by means of metabolic labelling in cell culture (Figure 1A). Specifically, we metabolically labeled A2780 ovarian cancer cells with ^13^C_6_^15^ N_4_-arginine and ^13^C_6_-lysine (heavy) for SILAC standard production. Non-labeled cell populations were instead grown in light medium (^12^C_6_^14^ N_4_-arginine and ^12^C_6_-lysine). After the complete incorporation of the “heavy” amino acids into the cells, A2780 “light” and “heavy” cells were transfected with a plasmid expressing eIF6 under the control of a strong promoter (hereafter termed as A2780/eIF6) and with the empty plasmid used as the standard (hereafter termed as “control”), respectively. Moreover, pEGFP plasmid was transfected in equal amounts in both of the previous transfections in order to detect the efficiency of DNA intake (Additional file 1: Figure S1). Following 48 h of growth, the transfected cells were analyzed by immunofluorescence. Those transfection assays showing a DNA intake higher than 60% were lysed and the effectiveness of eIF6 over-expression was verified by Western blotting (Figure 1B). The results of immunoblot and immunofluorescence experiments confirmed that A2780 cells received similar amounts of plasmid constructs in each transfection and that A2780/eIF6 cells displayed an increased expression of the ectopic protein, approximately two-fold with respect to the control.

For proteomics analysis, whole cell extracts isolated separately from “light” (empty vector) and “heavy” (eIF6 over-expression) cell lines were mixed in equal amounts. Then, the pooled sample was separated in membrane fraction enriched with integral and peripheral membrane-associated proteins (M fraction) with respect to the remaining “non-membranous” proteins defined as soluble cell fraction (S fraction). Next, both fractions were analyzed by Western blotting, investigating the presence of distinct markers characterizing the selective enrichment for the membrane proteins from A2780 cells (Figure 1C). The results of immune blots confirmed the accuracy of the cell fractioning procedure and permitted us to proceed to the proteomic analysis of the membrane fraction-associated proteins.

By means of nanoLC-MALDI-TOF/TOF analysis of two independent biological replicates we identified and quantified 576 proteins. Among them, we considered those proteins showing a SILAC ratio (Heavy/Light or Light/Heavy) ≥1.5 for subsequent analyses. By these criteria, in eIF6 over-expressing cells, 22 proteins were found down-regulated, while 66 showed an increased abundance (Additional file 2: Table S1).

### Interaction network generated by proteomic data highlights involvement of proteins entailed in cell migration

To address the biological relevance of the significantly and differentially regulated proteins following eIF6 over-expression, the proteomic data sets were further investigated *in silico* by Ingenuity Pathway Analysis (IPA) (Ingenuity Systems, Mountain View, CA; http://www.ingenuity.com). In particular, the web-based pathways analysis tool IPA allowed us to determine if proteins that changed in abundance could be mapped to specific functional networks that may be common to cell migration.

Table 1 shows that the enrichment results from the protein data set descends from an over-representation of genes related to high-level ontology database annotations of cell movement and migration of tumor cell lines (p-value of 4.49E-02 and 4.65E-02, respectively). In light of this, it is conceivable that the up-regulated proteins (i.e.: AGK, C1QBP, CDC42, HAX1, HGF, SDC1 and YBX1), involved in these biological functions, may be candidates as effectors of the eIF6-induced increased migration.

**Table 1:** **Biofunctional analysis by ingenuity pathway analysis**

Functions annotation	p-value	Predicted activation state	Activation z-score	Molecules
cell movement of tumor cell lines	4.49E-02	Increased	2.305	AGK,C1QBP,CDC42,HAX1,HGF,SDC1,YBX1
migration of tumor cell lines	4.65E-02	Increased	2.117	AGK,C1QBP,CDC42,HAX1,HGF,SDC1
cell death	4.85E-02	Decreased	-1.770	C1QBP,CD59,CDC42,COX5A,FDFT1,GAPDH,HAX1,HGF,HNRNPC,PGRMC1,RPS19,RTN4,SDC1,SLC25A4,TIMM50,YBX1

The genes up-regulated upon eIF6 overproduction mapped in a highly significant way to a functional network corresponding to cellular movement. Only data with significant Activation z-scores ≥ 1.5 or ≤ -1.5 were shown.

### Validation of changed cdc42 protein levels by western blotting

Successively, in order to uncover the actual participation of one of the above-predicted effectors on the increased cell migration we focused our attention on cdc42. Indeed, there is widely proven evidence in literature indicating that its enhanced activity is correlated to the augment of cell migration [20,21].

**Figure 2:**
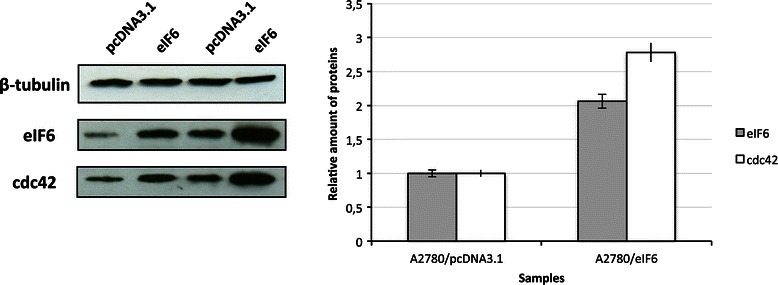
**eIF6 over-expression induces increased cdc42 protein levels in transiently transfected ovarian cancer cells.** cdc42 and eIF6 expression was analyzed by western blotting on the whole cell extracts of A2780 ovarian cancer cells. The bands were quantified by densitometry using the ImageJ software and the intensity of the protein bands was quantified relative to β-tubulin. The results represented in the histograms are shown as the mean ± S.D. and are the average of three independent experiments.

Preliminarily, we confirmed the proteomic results on the cdc42 differential expression by Western blotting. The analysis was performed on the whole cell extracts derived from other transfections replicating the experimental conditions adopted in the SILAC analysis (Figure 2). The results showed that the cdc42 up-regulation was in agreement with the data obtained by proteomic analysis. Moreover, the experiments performed on whole cell extracts highlighted genuine differential expression of the gene products instead of mere relocalization. Indeed, in the latter case the protein levels had to be unchanged.

### Increased amount of eIF6 perturbs cdc42 expression at the post-transcriptional level

Since eIF6 is characterized as a translation initiation factor, the most likely hypothesis is that it somehow differentially modulates the translation of the proteins involved in cell motility/invasiveness. However, we might speculate that the variation in abundance previously observed for some proteins is not directly controlled by eIF6 but rather by transcription factors or other transcriptional regulators which are under the direct control of eIF6 suggesting, as a consequence, an indirect effect of eIF6 on gene transcription of the differentially expressed target which was previously analyzed.

**Figure 3:**
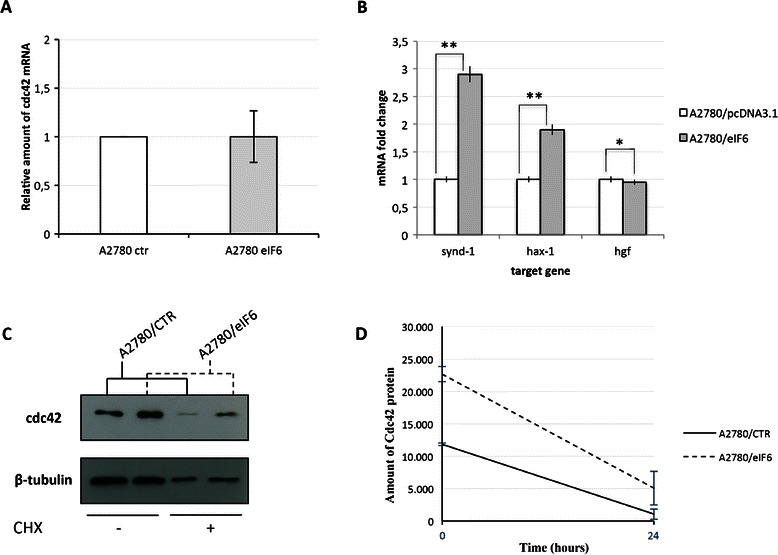
**The control of the increased cdc42 protein expression does not occur at the level of transcription or altered protein stability.** Analysis of differentially expressed mRNAs after increased eIF6 expression was performed on different target genes in A2780 ovarian cancer cells. **A)** qPCR of cdc42 mRNA was performed analysing 2 μg of total RNA reverse-transcribed into cDNA and comparing its expression between A2780 ovarian cancer cells over-expressing eIF6 with respect the control. The bar graphs represent the relative fold changes of cdc42 mRNA presented as mean ± S.D. and relative to that of GAPDH. The results are the average of three independent experiments. **B)** qPCR of synd-1, hax1 and hgf mRNA was performed analysing 1μg of total RNA reverse-transcribed into cDNA and comparing its expression between A2780 ovarian cancer cells over-expressing eIF6 with respect the control. The bar graphs represent the relative fold changes of target mRNAs presented as mean ± S.D. and relative to that of GAPDH. The results are the average of three independent experiments. The statistical analysis was performed with the *t*-test and the P-values were < 0.02 (**) and < 0.001 (*), respectively. **C-D)** To examine the stability of cdc42 protein, A2780 cells over-expressing eIF6 and the corresponding control were treated 24 hours after their transfection with 15 μM of the protein synthesis inhibitor CHX for the next 15 hours. Successively, endogenous cdc42 protein expression was detected by western blot analysis with an anti-cdc42 antibody and the intensity of the bands was normalized with respect the endogenous levels of β-tubulin. The expression levels of Cdc42 were determined by densitometry using ImageJ software. Results are shown for two of three independent experiments and are presented as mean ± S.D.

For this reason, we evaluated the transcriptional expression levels of cdc42 mRNA levels, using GADPH as an internal control. The quantitative RT-PCR did not show any difference of the cdc42 mRNA levels following eIF6 over-expression (Figure 3A). Noteworthy is the fact that the analysis of mRNAs expression levels for some of the other up-regulated proteins identified by IPA analysis upon eIF6 over-expression showed a real variation, suggesting, in this case, an indirect control of their expression by eIF6 (Figure 3B).

Moreover, in order to demonstrate that the changed levels of cdc42 protein did not arise from a differential control of its stability, we treated A2780 cells with cycloheximide (CHX). To this regard, A2780 cells were transfected with pcDNA3.1/eIF6 and *de novo* protein synthesis was blocked 24 h later with the translation inhibitor. Previous studies showed that the half-life of cdc42 was approximately 15 h [22]. For this reason, we extended the treatment of cells with CHX for the next 24 h after transfection. The results showed a turnover rate of cdc42 similar to the control (Figure 3C-D), suggesting that the increased expression of eIF6 does not induce a decreased protein turnover of cdc42 protein.

**Figure 4:**
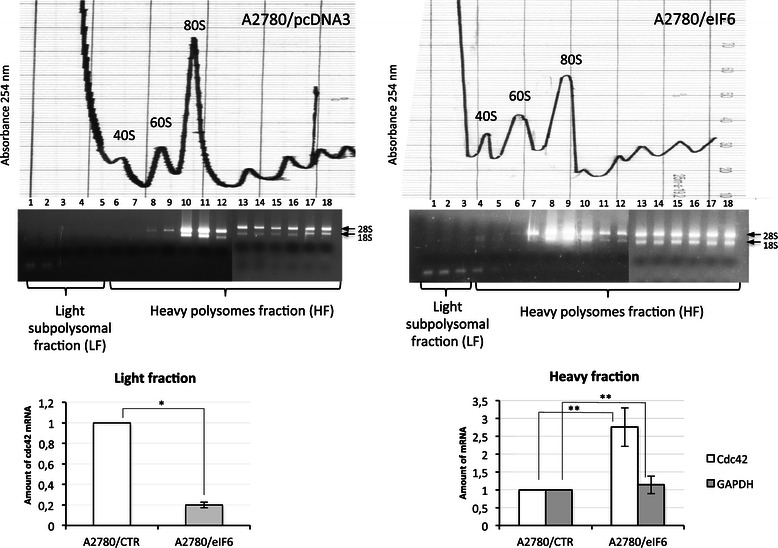
**eIF6 over-expression increased polysome loading of cdc42 mRNA.** The polysomal profiles of A2780/eIF6 and control cells were analysed by density gradient centrifugation. The sucrose gradient fractions were pooled together on the basis of the presence/absence of ribosomes, detected by ethidium bromide staining on agarose gels (upper panel). The total RNA of each polyribosomal fraction was extracted. Successively, cdc42 mRNA was measured in both fractions by RT-qPCR (bottom panel). The amount of cdc42 mRNA in the polysomal fractions was normalized using rRNA as the standard, while for ribosome-free fractions we used GAPDH mRNA levels. We also analysed GAPDH mRNA levels in the polysomal fractions normalizing with respect rRNA levels. The mean value is representative of three independent experiments with a P-value < 0.05 (**) and < 0.01 (*) respectively, calculated with the *t*-test.

Successively, in order to demonstrate that eIF6 overexpression influences translation of cdc42 mRNA, we measured the recruitment of cdc42 mRNA on polysomes by qRT–PCR. Indeed, as shown in Figure 4 eIF6 overexpression increased polysome loading of cdc42 mRNA with respect the total amount of rRNA, thereby suggesting that eIF6 impacts primarily on cdc42 translation.

### The enhanced levels of eIF6 induce cdc42 activation which in turn is accountable for increased cell migration

cdc42 is a small GTPase belonging to the Rho family that play major roles in regulating the actin cytoskeleton as well as key cellular functions such as differentiation, cell cycle progression, transformation, apoptosis, motility and adhesion. The activated form of cdc42 (cdc42-GTP) transmits signals by recruiting different proteins. Among these effectors are the p21-activated kinases (Paks) and serine/threonine kinases that also induce actin organization during cell adhesion and migration [23]. Moreover, ovarian cancer is characteristically metastatic and cdc42 has been speculated to be accountable for the migratory phenotype [24].

**Figure 5:**
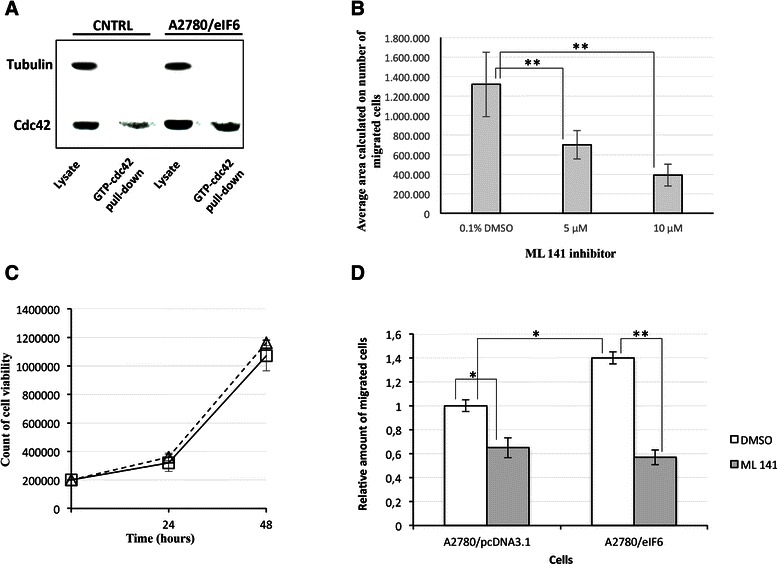
**Biochemical analysis of cdc42 activated form in A2780 ovarian cancer cells over-expressing eIF6. A)** Measurement of cdc42 activity analyzed by GST-PAK1 p21-binding domain pull-down. The figure shows one of three independent experiments with similar results. **B)** We treated A2780 cells (2.5 × 10^5^ per well) with the molecular probe ML 141 at the indicated concentrations for 72 hours. Mock treatments were carried out treating the cells in the same medium with DMSO 0,1%. Cells migrated in the lower chamber were stained with crystal violet dye. In the lower chamber, medium supplemented with 10% FBS was used as chemoattractant and also in this chamber the molecular probe was added at the concentration used in the upper chamber. The histograms are plotted as mean ± S.D. They represent the averages of three independent experiments with a P-value < 0.05 (**) calculated with the *t*-test. **C)** ML 141 did not show cytotoxicity in A2780 cell lines. The sensitivity was determined counting the number of cell viability by Trypan Blue exclusion staining. A2780 cells were treated with ML 141 10 μM or DMSO 0,1%. Cell viability was determined by trypan blue dye exclusion assay at the indicated time after ML 141 addition. The histograms represent the average of unstained cells and they are presented as mean ± S.D. The results assess three independent experiments. **D)** Enhanced migration of A2780 cells induced by eIF6 over-expression with respect the control cells was decreased in presence of cdc42 inhibitor ML 141. In particular, both control (pcDNA3.1) and eIF6-overexpressing (pcDNA3.1-eIF6) cells were affected in their migratory capacity by ML 141. However, the effect was more pronounced on A2780 cells over-expressing eIF6 for the synergistic effect of the inhibitor on both the intrinsic migratory capacity of the cells (as shown by the control) and the eIF6-induced motility.

Thus, we investigated whether eIF6 over-expression could induce the activation of cdc42-Pak signalling in A2780 ovarian cancer cells. Particularly, in order to detect the activation of cdc42 we used a recombinant cdc42-binding domain of PAK (PBD) that specifically binds and precipitates active GTP-bound cdc42. A2780 cells were lysed 24 h after their transfection with the appropriate constructs and the activated form of cdc42 was precipitated by GST fusion proteins of PBD, followed by Western blotting with an anti-cdc42 antibody (Cell Signalling). As shown in lane 3 of Figure 5A the enhanced expression of eIF6 induces an increased association and pull-down of active cdc42.

To further examine the role of the activated cdc42 form as an effector of increased cell migration in A2780 cells after eIF6 over-expression, we treated the cells with the molecular probe ML 141, a potent and selective inhibitor of cdc42 GTPase. It binds the guanine nucleotide-associated cdc42 and induces ligand dissociation [25]. Previous studies demonstrated that ML 141 inhibits the migration of human ovarian carcinoma cell lines OVCA429 and SKOV3 without exhibiting cytotoxicity [26]. However, since similar data for A2780 cells were not available, we preliminary treated A2780 cells with ML 141 in a dose-dependent manner. As shown in Figure 5B, we assayed the chemical compound at 5 and 10 μM, obtaining effective cell migration inhibition, even when using the smallest amount of the chemical. Moreover, ML 141 did not show cytotoxicity at the assayed concentration of 10 μM (Figure 5C). Successively, in order to verify whether the increased cell migration following eIF6 over-expression was cdc42-dependent, we probed the inhibitory effect of ML 141 in A2780 cells transfected with the specific constructs. To this end, transwell migration assays were performed with A2780 pCDNA3 control cells and A2780-eIF6 cells, in the presence of ML 141 inhibitor or its vehicle. As shown in Figure 5D, while the eIF6 over-expressing cells showed an increase in their capacity to pass through the matrigel layer, according to our previous data [16], the motility of both the A2780-pcDNA3.1 and the A2780-eIF6 cells was partially inhibited in the presence of cdc42 inhibitor. Notably, A2780-eIF6 cells showed a significantly more pronounced decrease in their migratory activity with respect to the control. Overall, these results suggest that cdc42 is clearly implicated in the control of cell motility induced by eIF6, although its inhibition is not sufficient to totally abrogate the acquired increased motility.

### eIF6 over-expression enhances cell migration, invasiveness and cdc42 protein expression in melanoma cell lines

To test whether the results of eIF6 over-expression on the migratory activity of the cells were generalizable to other cell line models, we extended our analysis on WM793 primary melanoma cell lines. Initially isolated from a superficial spreading melanoma presenting an early vertical growth phase, WM793 were considered poorly aggressive with a low metastatic potential with respect to the previously studied cell lines [27,28].

**Figure 6:**
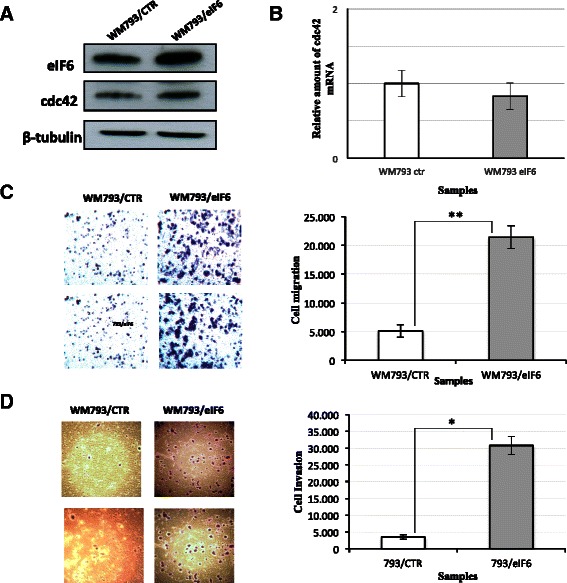
**eIF6 is implicated in the control of cell motility/invasiveness in WM793 melanoma cancer cells inducing an increased expression of cdc42 protein.** The results of eIF6 over-expression on the migratory activity of the cells were generalizable to other cell line models. **A)** eIF6 and cdc42 expression in WM793 primary melanoma cell lines transiently transfected either with the pcDNA3.1-eIF6 or control plasmid was analyzed by western blotting. **B)** qPCR of cdc42 mRNA was performed analysing 2 μg of total RNA reverse-transcribed into cDNA and comparing its expression between WM793 primary melanoma cell lines over-expressing eIF6 with respect the control. The bar graphs represent the relative fold changes of cdc42 mRNA presented as mean ± S.D. and relative to that of GAPDH. The results are the average of three independent experiments **C)** Migration assay: WM793/eIF6 and control cells were seeded in the upper side of migration chambers. The cells migrated to the lower chamber after 48 h of incubation were stained with crystal violet dye. **D)** Invasivity assay: cells were seeded in the upper side of invasion chambers. After 48 h cells migrated in the lower chamber were stained with crystal violet dye. The total stained area in the lower chambers was estimated using the Image-J software. The cell images in **C** and **D** are representative of three independent experiments. The histograms in **B**, **C** and **D** represent the average of three independent experiments. The P-values were calculated with the *t*-test using the symbols (**) and (*) corresponding to < 0.05 and < 0.01, respectively.

We transiently transformed WM793 melanoma cancer cell lines with the plasmid expressing eIF6. As shown in Figure 6A, the average expression of eIF6 did not exceed 2.5-fold its expression with respect to the control, similar to the previous results obtained with the A2780 cells. Moreover, we probed the same lysate samples with anti-cdc42 antibodies. The results confirmed an up-regulation of the protein to a similar extent of that observed in A2780 cells. Also in this case, the changed levels of cdc42 protein did not arise from a differential transcriptional control, as mRNA levels remained unchanged (Figure 6B).

According to our purpose, we tested whether eIF6 over-expression had any impact on the migratory and invasive capabilities of the WM793 cells. To this end, transwell migration and invasion assays were performed on WM793 transiently transfected with pcDNA3.1/eIF6 plasmid and the empty vector used as the control. As shown in Figure 6(C), the WM793/eIF6 cells displayed about a 4-fold increase in migratory capacity with respect to the WM793 cells transfected with the control. The most pronounced effects were obtained when the invasive capacity was tested by transwell/matrigel assays (Figure 6D). In this case the increased activity of invasion was about 6-fold higher than the control. This difference was greater with respect to the previous results obtained on A2780 cells [16], probably due to the poor basal invasive capacity of the WM793 cell lines rendering the eIF6-induced invasion activity more pronounced. Overall, the outcome of these experiments confirmed and extended the previous results observed in ovarian cancer cell lines, i.e. that eIF6 is implicated in the control of cell motility/invasiveness, also in different cellular contexts.

## Discussion

There is increasing evidence in the literature linking regulation of protein synthesis to cell transformation. For instance, it is well known that the altered expression of the translation initiation factor eIF4E contributes to cancer progression by enabling the translation of a limited pool of mRNAs encoding key proteins involved in cellular malignancy [7,29,30]. Similarly, the increased activity of translational initiation factors involved in the correct positioning of the pre-initiation complex (PIC) 43S on the first translatable codon AUG may cause the deregulation of signaling pathways causative of tumor progression.

Recently, the protein eIF6 has been added to the group of translation factors which are under the control of signal pathways sensing the nutrient levels of the surrounding environment. Specifically, the RACK1-PKC complex represents the last step of the Ras-PKC cascade, where phosphorylating eIF6 on Ser235 inhibits its association with 60S subunits. Moreover, eIF6 haploinsufficient mice are less susceptible to Myc and growth factor-induced tumors, suggesting that this protein is rate limiting for translation, cell growth and transformation [10].

In a previous work we observed that eIF6 over-expression in A2780 ovarian cancer cells stimulated their motility and invasiveness [16]. Here, we performed a proteomic analysis of membrane-associated proteins differentially expressed in cells transiently over-expressing eIF6. We focused our attention on the analysis of the membrane-bound proteins as those most likely to be affected by the pathways controlling cell motility and migration. In this regard, our analysis represents the first comprehensive overview of the impact of eIF6 over-expression on cellular membrane-bound proteins. Strikingly, we found that eIF6 over-expression in turn up-regulates a set of proteins participating in a functional network known to control tumor cell motility. Among these proteins, the most prominent were cdc42, syndecan-1, HCLS1-associated protein X-1 (also called HAX1) and the hepatocyte growth factor (HGF).

To confirm the validity of the proteomic analysis, we have further investigated the involvement in eIF6-induced motility of cdc42, a member of the Rho GTPase subfamily, known to be involved in actin cytoskeletal reorganization, cell adhesion, cell migration, invasion, and control of cell cycle progression. We found that besides up-regulating the levels of cdc42, eIF6 over-expression increased the amount of active cdc42 forms (GTP-bound), thereby stimulating the cdc42-Pak signalling. Moreover, we demonstrated that the use of the specific cdc42 inhibitor ML-141 decreased eIF6 induced cell migration. Finally, over-expression of eIF6 in primary melanoma cell lines (WM793) induced cdc42 up-regulation and increased motility and invasiveness, thus demonstrating that the tumor-promoting ability of this initiation factor is not restricted to the A2780 cell line. Notably, both eIF6 and cdc42 have been reported to be up-regulated in cells over-expressing PRL-1, a putative oncogene involved in the control of a number of diverse biological processes, including migration and invasion [31]. Our results could suggest a possible mechanism of regulation in which eIF6 act as a mediator of the cdc42 expression at the translational level, although additional experiments are needed to elucidate this issue.

However, besides cdc42, further investigation is required to gain deep insights on the molecular mechanisms by which eIF6 overexpression promotes cell migration. In this regard, both the precise role of the other membrane-associated main targets of eIF6 (syndecan-1, HCLS1-associated protein X-1, HGF) and the extension of the proteomic analysis on soluble proteins need to be defined. The importance of this analysis is also indicated by the fact that some of the proteins up-regulated upon eIF6 over-expression had altered steady state mRNA levels, suggesting, in this case, an indirect control of their expression by eIF6, possibly via the translational modulation of some transcription factor.

eIF6 was originally described as a ribosome anti-association factor, and indeed it has a dose-dependent inhibitory effect on *in vitro* translation [32,33]. *In vivo*, variations in eIF6 abundance do not seem to grossly affect global protein synthesis [16,12]. However, it must be borne in mind that viable transformed cells displayed, at most, a two-three-fold over-expression of the protein, thus suggesting that high amounts of eIF6 are lethal. In the light of these data, the most probable hypothesis is that eIF6 overabundance alters the rate/efficiency at which certain mRNAs are translated, favouring up-regulation of motility-promoting proteins that are normally poorly translated.

The main difficulty in comprehending the mechanism whereby this alteration of the translational landscape may take place is that the function of eIF6 in translation is not completely understood. A number of data indicate that the factor may participate in ribosome recycling [34]. If this is true, an excess of eIF6 may increase recycling, perhaps making more ribosomes available for the translation of certain mRNAs. There is also evidence that eIF6 is involved in ribosome biogenesis [35,36]. In this capacity, an excess of the factor may produce altered ribosomes that may bind certain mRNA classes preferentially. Some evidence in support of the latter idea comes from our quantitative proteomic analysis, which showed that eIF6 over-expression also affects the abundance of certain RPs in membrane-associated ribosomes (Additional file 2: Table S1). Strikingly, some of these RPs are located on 60S subunits mapping in the vicinity of the eIF6 binding site (RPL13a, RPL24 and RPL35a), suggesting a common functional activity. The idea that perturbations in ribosome structure may deregulate translation of mRNAs encoding cancer-promoting proteins is supported by published data, as illustrated by X-linked dyskeratosis congenita [37] or from research performed on single mutated genes coding for RPs, as RPL38 or RPL10 [38,39].

## Conclusion

In conclusion, our results contribute to shed light on the role of eIF6 in the onset and progression of cell transformation, thus suggesting a molecular platform for developing new anti-cancer strategies.

## Additional files

Additonal file 1: Figure S1. Analysis of transfected cells.
Additional file 2: Table S1. Membrane associated proteins expression levels in A2780/eIF6 vs A2780/pcDNA3.1 cells, as identified by nanoLC-MS/MS analysis. Significant (p≤0,05) differentially expressed proteins with fold change higher then 1,5 are reported.

## Abbreviations

SILAC: Stable isotope labeling by/with amino acids in cell culture
Paks: p21-activated kinases
IPA: Ingenuity pathway analysis
CHX: Cycloheximide

## References

[1] Conlon I, Raff M (1999). Size control in animal development. Cell.

[2] Derenzini M, Ploton D (1991). Interphase nucleolar organizer regions in cancer cells. Int Rev Exp Pathol.

[3] Spriggs KA, Bushell M, Willis AE (2010). Translational regulation of gene expression during conditions of cell stress. Mol Cell.

[4] Mayer C, Grummt I (2006). Ribosome biogenesis and cell growth: mTOR coordinates transcription by all three classes of nuclear RNA polymerases. Oncogene.

[5] Rajasekhar VK, Holland EC (2004). Postgenomic global analysis of translational control induced by oncogenic signalling. Oncogene.

[6] Graff JR, Konicek BW, Lynch RL, Dumstorf CA, Dowless MS, McNulty AM (2009). eIF4E activation is commonly elevated in advanced human prostate cancers and significantly related to reduced patient survival. Cancer Res.

[7] Graff JR, Zimmer SG (2003). Translational control and metastatic progression: enhanced activity of the mRNA cap-binding protein eIF-4E selectively enhances translation of metastasis-related mRNAs. Clin Exp Metastasis.

[8] De Benedetti A, Graff JR (2004). eIF-4E expression and its role in malignancies and metastases. Oncogene.

[9] Freed EF, Bleichert F, Dutca LM, Baserga SJ (2010). When ribosomes go bad: diseases of ribosome biogenesis. Mol Biosyst.

[10] Gandin V, Miluzio A, Barbieri AM, Beugnet A, Kiyokawa H, Marchisio PC (2008). Eukaryotic initiation factor 6 is rate-limiting in translation, growth and transformation. Nature.

[11] Rosso P, Cortesina G, Sanvito F, Donadini A, Di Benedetto B, Biffo S (2004). Overexpression of p27BBP in head and neck carcinomas and their lymph node metastases. Head Neck.

[12] Miluzio A, Beugnet A, Grosso S, Brina D, Mancino M, Campaner S (2011). Impairment of cytoplasmic eIF6 activity restricts lymphomagenesis and tumor progression without affecting normal growth. Cancer Cell.

[13] Groft CM, Beckmann R, Sali A, Burley SK (2000). Crystal structures of ribosome anti-association factor IF6. Nat Struct Biol.

[14] Russell DW, Spremulli LL (1979). Purification and characterization of a ribosome dissociation factor (eukaryotic initiation factor 6) from wheat germ. J Biol Chem.

[15] Basu U, Si K, Warner JR, Maitra U (2001). The Saccharomyces cerevisiae TIF6 gene encoding translation initiation factor 6 is required for 60S ribosomal subunit biogenesis. Mol Cell Biol.

[16] Benelli D, Cialfi S, Pinzaglia M, Talora C, Londei P (2012). The translation factor eIF6 is a notch-dependent regulator of cell migration and invasion. PLoS One.

[17] Talora C, Campese AF, Bellavia D, Felli MP, Vacca A, Gulino A (2008). Notch signaling and diseases: an evolutionary journey from a simple beginning to complex outcomes. Biochim Biophys Acta.

[18] Hsieh AC, Liu Y, Edlind MP, Ingolia NT, Janes MR, Sher A (2012). The translational landscape of mTOR signalling steers cancer initiation and metastasis. Nature.

[19] Zhu LJ, Altmann SW (2005). mRNA and 18S-RNA coapplication-reverse transcription for quantitative gene expression analysis. Anal Biochem.

[20] Yamazaki D, Kurisu S, Takenawa T (2005). Regulation of cancer cell motility through actin reorganization. Cancer Sci.

[21] Bray K, Gillette M, Young J, Loughran E, Hwang M, Sears JC (2013). Cdc42 overexpression induces hyperbranching in the developing mammary gland by enhancing cell migration. Breast Cancer Res.

[22] Backlund PS (1997). Post-translational processing of RhoA carboxyl methylation of the carboxyl-terminal prenylcysteine increases the half-life of Rhoa. J Biol Chem.

[23] Kiosses WB, Daniels RH, Otey C, Bokoch GM, Schwartz MA (1999). A role for p21-activated kinase in endothelial cell migration. J Cell Biol.

[24] Ip CK, Cheung AN, Ngan HY, Wong AS (2011). p70 S6 kinase in the control of actin cytoskeleton dynamics and directed migration of ovarian cancer cells. Oncogene.

[25] Surviladze Z, Waller A, Strouse JJ, Bologa C, Ursu O, Salas V (2010). A Potent and Selective Inhibitor of Cdc42 GTPase. Probe Reports from the NIH Molecular Libraries Program.

[26] Hong L, Kenney SR, Phillips GK, Simpson D, Schroeder CE, Nöth J (2013). Characterization of a Cdc42 protein inhibitor and its use as a molecular probe. J Biol Chem.

[27] Hsu MY, Elder DE, Herlyn M, Masters JRW, Palsson B (1999). Melanoma: The Wistar (WM) Melanoma Cell Lines. Human Cell Culture.

[28] Kobayashi H, Man S, MacDougall JR, Graham CH, Lu C, Kerbel RS (1994). Variant sublines of early-stage human melanomas selected for tumorigenicity in nude mice express a multicytokine-resistant phenotype. Am J Pathol.

[29] Gallagher WM, Bergin OE, Raffertyy M, Kellyy ZD, Nolan IM, Fox E (2005). Multiple markers for melanoma progression regulated by DNA methylation: insights from transcriptomic studies. Carcinogenesis.

[30] Mamane Y, Petroulakis E, Rong L, Yoshida K, Wee Ler L, Sonenberg N (2004). eIF4E – from translation to transformation. Oncogene.

[31] Dumaual CM, Steere BA, Walls CD, Wang M, Zhang ZY, Randall SK (2013). Integrated analysis of global mRNA and protein expression data in HEK293 cells overexpressing PRL-1. PLoS One.

[32] Stumpf CR, Ruggero D (2011). The cancerous translation apparatus. Curr Opin Genet Dev.

[33] Dario Benelli D, Marzi S, Mancone C, Alonzi T, La Teana A, Londei P (2009). Function and ribosomal localization of aIF6, a translational regulator shared by archaea and eukarya. Nucleic Acids Res.

[34] Ceci M, Gaviraghi C, Gorrini C, Sala LA, Offenhäuser N, Marchisio PC (2003). Release of eIF6 (p27BBP) from the 60S subunit allows 80S ribosome assembly. Nature.

[35] Pisarev AV, Skabkin MA, Pisareva VP, Skabkina OV, Rakotondrafara AM, Hentze MW (2010). The role of ABCE1 in eukaryotic post-termination ribosomal recycling. Mol Cell.

[36] Wong CC, Traynor D, Basse N, Kay RR, Warren AJ (2011). Defective ribosome assembly in Shwachman-Diamond syndrome. Blood.

[37] Yoon A, Peng G, Brandenburger Y, Zollo O, Xu W, Rego E (2006). Impaired control of IRES-mediated translation in X-linked dyskeratosis congenita. Science.

[38] Kondrashov N, Pusic A, Stumpf CR, Shimizu K, Hsieh AC, Xue S (2011). Ribosome-mediated specificity in Hox mRNA translation and vertebrate tissue patterning. Cell.

[39] Sulima SO, Patchett S, Advani VM, De Keersmaecker K, Johnson AW, Dinman JD (2014). Bypass of the pre-60S ribosomal quality control as a pathway to oncogenesis. Proc Natl Acad Sci U S A.

