# Supplementary figures and images for: eIF6 over-expression increases the motility and invasiveness of cancer cells by modulating the expression of a critical subset of membrane-bound proteins
Source: BMC Cancer. 2015 Mar 15;15:131. doi: 10.1186/s12885-015-1106-3 (PMC4381359; doi:10.1186/s12885-015-1106-3)

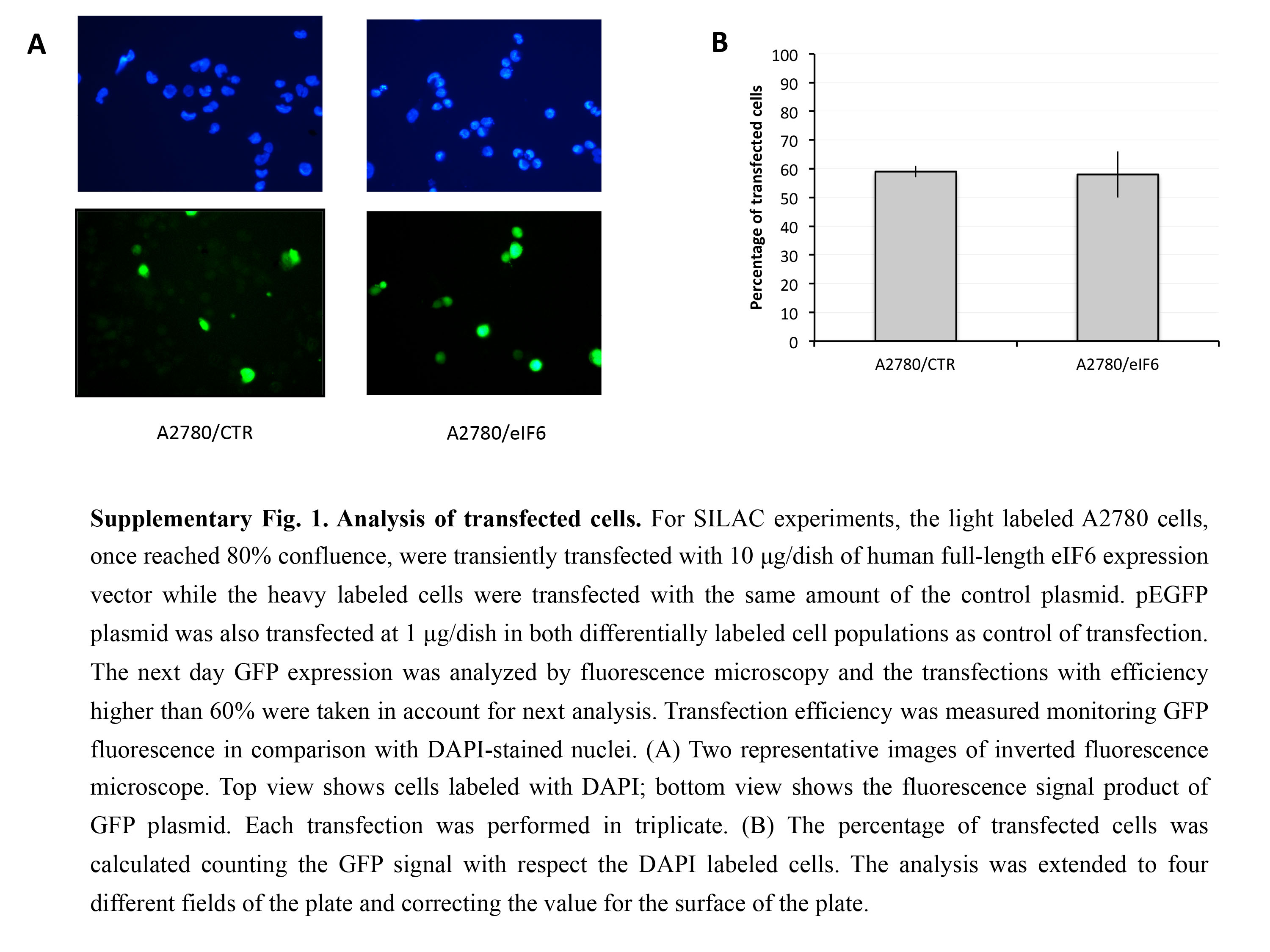

Supplement: Additonal file 1: Figure S1. — Analysis of transfected cells. [file 12885_2015_1106_MOESM1_ESM.jpeg]
