# Supplementary material for: eIF6 over-expression increases the motility and invasiveness of cancer cells by modulating the expression of a critical subset of membrane-bound proteins
Source: BMC Cancer. 2015 Mar 15;15:131. doi: 10.1186/s12885-015-1106-3 (PMC4381359; doi:10.1186/s12885-015-1106-3)
Supplement: Additional file 2: Table S1. — Membrane associated proteins expression levels in A2780/eIF6 vs A2780/pcDNA3.1 cells, as identified by nanoLC-MS/MS analysis. Significant (p≤0,05) differentially expressed proteins with fold change higher then 1,5 are reported. [file 12885_2015_1106_MOESM2_ESM.doc]

**Supplementary Table 1 Membrane associated proteins expression levels in A2780/eIF6 vs A2780/pcDNA3.1 cells, as identified by nanoLC-MS/MS analysis. Significant (p≤0,05) differentially expressed proteins with fold change higher then 1,5 are reported.**

| **Acc. number^a^** | **Protein name** | **Light(eiF6)/Heavy(Ctr) ratio** | **Fold Change Light (eIF6) in respect to Heavy (Ctr)** |
| --- | --- | --- | --- |
| P40429 | 60S ribosomal protein L13a | 0,44 | -2,28 |
| Q9BYN8 | 28S ribosomal protein S26, mitochondrial | 0,45 | -2,21 |
| P26440 | Isovaleryl-CoA dehydrogenase, mitochondrial | 0,49 | -2,02 |
| P13987 | CD59 glycoprotein | 0,58 | -1,73 |
| P46778 | 60S ribosomal protein L21 | 0,59 | -1,69 |
| P63162 | Small nuclear ribonucleoprotein-associated protein N | 0,60 | -1,66 |
| P62805 | Histone H4 | 0,61 | -1,65 |
| Q9Y277 | Voltage-dependent anion-selective channel protein 3 | 0,62 | -1,60 |
| Q12797 | Aspartyl/asparaginyl beta-hydroxylase | 0,63 | -1,59 |
| O95299 | NADH dehydrogenase [ubiquinone] 1 alpha subcomplex subunit 10, mitochondrial | 0,63 | -1,59 |
| O15260 | Surfeit locus protein 4 | 0,64 | -1,57 |
| P83731 | 60S ribosomal protein L24 | 0,64 | -1,56 |
| Q8TCJ2 | Dolichyl-diphosphooligosaccharide--protein glycosyltransferase subunit STT3B | 0,64 | -1,56 |
| P18077 | 60S ribosomal protein L35a | 0,65 | -1,55 |
| P53007 | Tricarboxylate transport protein, mitochondrial | 0,65 | -1,54 |
| P11310-2 | Isoform 2 of Medium-chain specific acyl-CoA dehydrogenase, mitochondrial | 0,65 | -1,53 |
| P04406 | Glyceraldehyde-3-phosphate dehydrogenase | 0,66 | -1,52 |
| P12235 | ADP/ATP translocase 1 | 0,66 | -1,52 |
| P62081 | 40S ribosomal protein S7 | 0,66 | -1,52 |
| P09669 | Cytochrome c oxidase subunit 6C | 0,66 | -1,51 |
| P39019 | 40S ribosomal protein S19 | 0,66 | -1,51 |
| Q15363 | Transmembrane emp24 domain-containing protein 2 | 0,66 | -1,50 |
| Q15365 | Poly(rC)-binding protein 1 | 1,50 | 1,50 |
| P04181 | Ornithine aminotransferase, mitochondrial | 1,50 | 1,50 |
| P38117-2 | Isoform 2 of Electron transfer flavoprotein subunit beta | 1,51 | 1,51 |
| Q15691 | Microtubule-associated protein RP/EB family member 1 | 1,52 | 1,52 |
| Q07021 | Complement component 1 Q subcomponent-binding protein, mitochondrial | 1,53 | 1,53 |
| P30042 | ES1 protein homolog, mitochondrial | 1,54 | 1,54 |
| P56385 | ATP synthase subunit e, mitochondrial | 1,54 | 1,54 |
| Q96AG4 | Leucine-rich repeat-containing protein 59 | 1,54 | 1,54 |
| P14210 | Hepatocyte growth factor | 1,56 | 1,56 |
| P82673 | 28S ribosomal protein S35, mitochondrial | 1,56 | 1,56 |
| Q96DV4 | 39S ribosomal protein L38, mitochondrial | 1,59 | 1,59 |
| P63261 | Actin, cytoplasmic 2 | 1,60 | 1,60 |
| P13995 | Bifunctional methylenetetrahydrofolate dehydrogenase/cyclohydrolase, mitochondrial | 1,60 | 1,60 |
| Q5RI15 | Protein FAM36A | 1,60 | 1,60 |
| Q5SRD1 | Putative mitochondrial import inner membrane translocase subunit Tim23B | 1,62 | 1,62 |
| Q9BXW7 | Cat eye syndrome critical region protein 5 | 1,62 | 1,62 |
| P42765 | 3-ketoacyl-CoA thiolase, mitochondrial | 1,62 | 1,62 |
| P49753 | Acyl-coenzyme A thioesterase 2, mitochondrial | 1,63 | 1,63 |
| Q96C36 | Pyrroline-5-carboxylate reductase 2 | 1,65 | 1,65 |
| Q9NR30 | Nucleolar RNA helicase 2 | 1,67 | 1,67 |
| Q6ZRP7 | Sulfhydryl oxidase 2 | 1,69 | 1,69 |
| O75439 | Mitochondrial-processing peptidase subunit beta | 1,70 | 1,70 |
| Q14257 | Reticulocalbin-2 | 1,72 | 1,72 |
| P07910-2 | Isoform C1 of Heterogeneous nuclear ribonucleoproteins C1/C2 | 1,72 | 1,72 |
| P51991 | Heterogeneous nuclear ribonucleoprotein A3 | 1,72 | 1,72 |
| Q9NZ01 | Trans-2,3-enoyl-CoA reductase OS=Homo sapiens GN=TECR PE=1 SV=1 | 1,72 | 1,72 |
| Q16643 | Drebrin | 1,72 | 1,72 |
| P11047 | Laminin subunit gamma-1 | 1,72 | 1,72 |
| P08574 | Cytochrome c1, heme protein, mitochondrial | 1,76 | 1,76 |
| P38117 | Electron transfer flavoprotein subunit beta | 1,78 | 1,78 |
| Q5JPE7 | Nodal modulator 2 | 1,78 | 1,78 |
| O00264 | Membrane-associated progesterone receptor component 1 | 1,79 | 1,79 |
| P61966 | AP-1 complex subunit sigma-1A | 1,79 | 1,79 |
| Q9HAV7 | GrpE protein homolog 1, mitochondrial | 1,80 | 1,80 |
| O95169 | NADH dehydrogenase [ubiquinone] 1 beta subcomplex subunit 8, mitochondrial | 1,86 | 1,86 |
| Q96A35 | 39S ribosomal protein L24, mitochondrial | 1,87 | 1,87 |
| P00403 | Cytochrome c oxidase subunit 2 | 1,92 | 1,92 |
| P62424 | 60S ribosomal protein L7a | 1,93 | 1,93 |
| Q53H12 | Acylglycerol kinase, mitochondrial | 1,97 | 1,97 |
| Q8NBQ5 | Estradiol 17-beta-dehydrogenase 11 | 1,99 | 1,99 |
| Q3ZCQ8-2 | Isoform Tim50a of Mitochondrial import inner membrane translocase subunit TIM50 | 2,03 | 2,03 |
| Q9Y3B7 | 39S ribosomal protein L11, mitochondrial | 2,05 | 2,05 |
| P37268 | Squalene synthase | 2,06 | 2,06 |
| P20674 | Cytochrome c oxidase subunit 5A, mitochondrial | 2,08 | 2,08 |
| P67809 | Nuclease-sensitive element-binding protein 1 | 2,08 | 2,08 |
| P62988 | Ubiquitin | 2,09 | 2,09 |
| Q07020 | Fatty acid desaturase 2 | 2,12 | 2,12 |
| O00165 | 40S ribosomal protein S15 | 2,12 | 2,12 |
| O00217 | Isoform BOV-1b of RNA-binding protein 8A | 2,14 | 2,14 |
| Q9NWU5 | 60S ribosomal protein L18 | 2,19 | 2,19 |
| P01889 | HCLS1-associated protein X-1 | 2,26 | 2,26 |
| Q9NRP0 | NADH dehydrogenase [ubiquinone] iron-sulfur protein 8, mitochondrial | 2,26 | 2,26 |
| Q96HS1 | 39S ribosomal protein L22, mitochondrial | 2,43 | 2,43 |
| O75976 | HLA class I histocompatibility antigen, B-7 alpha chain | 2,50 | 2,50 |
| P10321 | Oligosaccharyltransferase complex subunit OSTC | 2,54 | 2,54 |
| Q9BYD3 | Serine/threonine-protein phosphatase PGAM5, mitochondrial | 2,58 | 2,58 |
| Q9Y2Q3 | Carboxypeptidase D | 2,59 | 2,59 |
| Q6UW68 | HLA class I histocompatibility antigen, Cw-7 alpha chain | 2,74 | 2,74 |
| P60953-2 | 39S ribosomal protein L4, mitochondrial | 2,75 | 2,75 |
| Q9NQC3-2 | Glutathione S-transferase kappa 1 | 2,75 | 2,75 |
| P18827 | Transmembrane protein 205 | 2,97 | 2,97 |
| P60953-2 | Cell division control protein 42 homolog | 3,2 | 3,2 |
| Q9NQC3-2 | Reticulon-4 | 3,29 | 3,29 |
| P18827 | Syndecan-1 | 3,89 | 3,89 |
| P07910 | Heterogeneous nuclear ribonucleoproteins C1/C2 | 4,18 | 4,18 |

^a^According to the UniProtKB/Swiss-Prot entry
